# Formulations for Intranasal Delivery of Pharmacological Agents to Combat Brain Disease: A New Opportunity to Tackle GBM?

**DOI:** 10.3390/cancers5031020

**Published:** 2013-08-14

**Authors:** Matthias van Woensel, Nathalie Wauthoz, Rémi Rosière, Karim Amighi, Véronique Mathieu, Florence Lefranc, Stefaan W. van Gool, Steven de Vleeschouwer

**Affiliations:** 1 Laboratory of Experimental Neurosurgery and Neuroanatomy, KU Leuven, Leuven 3000, Belgium; E-Mail: steven.devleeschouwer@uzleuven.be; 2 Laboratory of Pediatric Immunology, KU Leuven, Leuven 3000, Belgium; E-Mail: stefaan.vangool@uzleuven.be; 3 Laboratory of Pharmaceutics and Biopharmaceutics, ULB, Brussels 1050, Belgium; E-Mails: nawautho@ulb.ac.be (N.W.); rrosiere@ulb.ac.be (R.R.); kamighi@ulb.ac.be (K.A.); 4 Laboratory of Toxicology, ULB, Brussels 1050, Belgium; E-Mails: vemathie@ulb.ac.be (V.M.); florence.lefranc@erasme.ulb.ac.be (F.L.); 5 Department of Neurosurgery, Erasmus University Hospitals, Brussels 1050, Belgium; 6 Department of Neurosurgery, University Hospitals Leuven, Leuven 3000, Belgium

**Keywords:** glioblastoma multiforme, intranasal administration, nose-to-brain delivery, formulations, nanoparticles, drug delivery, new therapy concept

## Abstract

Despite recent advances in tumor imaging and chemoradiotherapy, the median overall survival of patients diagnosed with glioblastoma multiforme does not exceed 15 months. Infiltration of glioma cells into the brain parenchyma, and the blood-brain barrier are important hurdles to further increase the efficacy of classic therapeutic tools. Local administration methods of therapeutic agents, such as convection enhanced delivery and intracerebral injections, are often associated with adverse events. The intranasal pathway has been proposed as a non-invasive alternative route to deliver therapeutics to the brain. This route will bypass the blood-brain barrier and limit systemic side effects. Upon presentation at the nasal cavity, pharmacological agents reach the brain via the olfactory and trigeminal nerves. Recently, formulations have been developed to further enhance this nose-to-brain transport, mainly with the use of nanoparticles. In this review, the focus will be on formulations of pharmacological agents, which increase the nasal permeation of hydrophilic agents to the brain, improve delivery at a constant and slow release rate, protect therapeutics from degradation along the pathway, increase mucoadhesion, and facilitate overall nasal transport. A mounting body of evidence is accumulating that the underexplored intranasal delivery route might represent a major breakthrough to combat glioblastoma.

## 1. Introduction to Glioblastoma

### 1.1. Epidemiology

Gliomas are by far the most common type of intrinsic brain tumor in adults, affecting 5–10 individuals/100,000/year, and account for more than 50% of all intrinsic brain tumors. Histopathologically, gliomas can be subtyped according to their nature of origin: astrocytomas (60%–70%), oligodendroglial tumors (10%–30%), ependymal tumors (<10%), and mixed gliomas [1]. Within the group of astrocytic tumors, high grade gliomas are more common. Glioblastoma multiforme (GBM), a grade IV astrocytoma, is approximately four times more common than grade III anaplastic astrocytoma [2]. Despite a standard multimodal treatment for newly diagnosed GBM, consisting of maximal safe neurosurgical resection, radiation, and chemotherapy, the prognosis remains dismal, with a median survival of 14.6 months [3].

### 1.2. Standard of Care

Diagnosis of GBM is a multidisciplinary exercise for clinicians. Clinical symptoms can present as seizures, headaches and focal neurological deficits that correlate with the tumor-site such as aphasia, motor and sensibility deficits. Cognitive dysfunction is also extremely common in malignant gliomas. When a brain tumor is suspected, magnetic resonance imaging (MRI), with or without contrast infusion is preferred. Tumor imaging is indispensible for maximal safe neurosurgical resection, for high grade gliomas, preferentially with 5-aminolevulinic acid for intra-operative real-time fluorescence guidance [4]. After corticosteroid treatment, to reduce the vasogenic edema, and after resection, patients enter the “Stupp-regimen” [3]. Patients receive radio- and chemotherapy, temozolomide (TMZ), concomitant and later as adjuvant chemotherapy. This treatment results in the current prognosis of a median survival of 14.6 months from diagnosis.

### 1.3. Shortcomings of Standard Care and Novel Treatments

#### 1.3.1. Tumor Vaccines

Due to the dismal prognosis, which is a result of the local aggressiveness and therapy resistance of GBM, reflected in the highest number of years of loss of life [5], the highest disability and need for care, new therapies are absolutely needed. Immunotherapy with dendritic cell (DC)-based vaccinations and peptide vaccinations are currently under intensive investigation [6]. These active immunotherapy methods target the patient’s own immune system towards the tumor by administration of an autologous vaccine or immunogenic peptides. In case of DC-based vaccination, DCs are generated *ex vivo* and loaded with tumor antigens aimed to stimulate the adoptive immune system [7]. Our research group has established several clinical trials with beneficial therapeutic effects, both in combination therapy with the Stupp-regimen, as in postoperative adjuvant therapy in recurrent setting. In a large group of relapsed GBM patients, we reported a substantial amount of long term survivors, surviving more than 24 months after re-operation [8]. In newly diagnosed GBM patients, we reported a shift in median survival from 14.6 months towards 18.3 months, and a two year survival rate of more than 42% of the patients according to long-term analysis data. Furthermore, these studies demonstrated the safety and feasibility of the DC-based vaccination strategy [9,10]. As with many other treatment modalities, it remains puzzling why active immunotherapy by means of DC-based vaccinations does not elicit clinical responses in all patients.

#### 1.3.2. Local Administration

As the brain is well-protected by the blood-brain barrier (BBB), administering pharmacological agents with intracerebral biological activity is a challenging task [11]. Even when drugs are permeable through the BBB, it is often difficult to reach therapeutic intratumoral concentrations. To overcome this problem, local administrations can be used, such as convection enhanced delivery (CED), intracerebral infusion, and wafers at the resection site. CED is a continuous infusion that uses a convective flow to drive the pharmacological agent into a large tissue area. One or more catheters are placed in the tumor mass, around the tumor or the resection cavity. Even with the use of well-developed catheters as reflux-preventing, hollow-fiber and balloon-tipped catheters, the leakage of infusates is nearly always detected [12]. This leakage is a waste of therapeutic agent, and can, moreover, exert possible adverse effects on the healthy surrounding tissue and complicates the ability to estimate reliable iso-distribution volumes. Also, for CED, encouraging clinical data have been obtained, for example in the field of cytotoxin administration via CED [13]. Cytotoxins are recombinantly made proteins that consist of a vector/ligand/receptor and a bacterial toxin. A first example was DT.CRM107-Tf, a conjugate between transferrin and a diphtheria toxin derivative. Several clinical responses were observed but in phase III, toxicity was observed and the trial was stopped [14]. CED administration of IL13-PE38QQR, a cytotoxin that consists of IL-13 and Pseudomonas exotoxin A, was evaluated in a randomized phase III trial with recurrent GBM patients. It showed a median overall survival of 42.7 weeks and even up to 55.6 weeks for patients with an optimally positioned catheter [15]. The local administration of chemotherapeutic drugs via CED, for example taxol, has also been explored in brain tumor patients and seems to have modest beneficial effects [16]. We refer to an excellent review by Debinski *et al.* for additional information on the topic of CED in the context of brain tumors [17]. Despite these positive clinical outcomes, complications are unfortunately inherent to CED. Surgical installation of one or more catheters, and the convective inward flow, often lead to complications such as infection, wound healing problems, inflammation, edema, and seizures [18,19]. In particular, CED is unlikely to be practical for drugs which need to be administered chronically. In more recent years, the intranasal pathway has being discovered as a non-invasive alternative to the invasive CED treatment modality.

## 2. Intranasal Administration

Intranasal transport is the direct transport of therapeutic agents from the nasal cavity to the brain. This is a mainly extracellular and transcellular transport, involving the olfactory and respiratory regions of the nasal cavity. Intranasal administration has already been used for many years in the clinic to administer substances that cannot be given orally. These substances will reach the systemic circulation through intranasal instillation. Only a few decades ago, the potential of intranasal administration to reach the central nervous system (CNS) gained interest [20,21]. Pharmacological agents can bypass the BBB during this transport and enter the CNS. The BBB is normally only permeable to lipophilic molecules, with a molecular weight (Mw) less than 600 Dalton (Da) [22]. The LogP, the partition coefficient between solubility in octanol versus water, is estimated to be 1.5–2.7 for an efficient transport over the BBB [23]. The very low permeability of the BBB is associated with low levels of pinocytosis and the presence of tight-junctions (TJs), which is critical for the CNS to maintain homeostasis [24]. Furthermore the BBB is also equipped with a high number of drug transporters, such as P-glycoprotein (P-gp), which further prevents the entry of pharmacological drugs to the CNS [25]. By circumventing the BBB via intranasal transport, the repertoire of possible therapeutic agents can be expanded to proteins, cells, nucleotides, viral vectors, and chemotherapeutics. Moreover, advantages of nose-to-brain transport include the avoidance of the systemic circulation, reducing the risk of systemic side effects and hepatic/renal clearing, and the possibility of chronic administration. Its non-invasiveness, the self-administration by patients with high patient compliance, and the rapid onset of action represent an attractive option to further explore this route of administration to ultimately improve the prognosis for GBM patients.

## 3. Anatomy Relevant for Nose-to-Brain Transport

In the next paragraph the anatomical organization of the nasal cavity will be discussed, in particular the structures that are necessary for understanding nose-to-brain transport. This topic has already been the subject of several excellent reviews [22,26,27]. Therefore only a summary is present here, underlining the key-points in the anatomical organization in the nasal cavity relevant for intranasal transport to the CNS. First, the possible pathways that are responsible for an effective nose-to-brain transport will be discussed. Next, a closer look will be provided into the anatomical structures that decide whether the applied substance can undergo nose-to-brain transport.

### 3.1. Macroscopical Anatomy

#### 3.1.1. Olfactory Pathway/Olfactory Region (Figure 1A)

The exact mechanisms underlying nose-to-brain transport are not yet fully understood, but the olfactory pathway seems to play a pronounced role. The olfactory region in humans accounts for <10% of the nasal cavity. Pharmaceutical agents can gain fast access to the CNS along the olfactory nerve fibers of the olfactory bulb, which is the only anatomical structure of the CNS that is in direct physical contact with the environment. This was well-illustrated by Jansson *et al.* by intranasal administration of a fluorescent dye and subsequently monitoring of the route of transport along the olfactory nerves [28]. The olfactory pathway starts at the olfactory receptor neurons, located at the olfactory mucosa. These cells pick up olfactants and transmit the information to the CNS, mediating the sense of smell [29]. The olfactory receptor neurons are surrounded by basal cells, microvillar cells, and supporting cells, all connected by TJs. These basal cells act as neural progenitors cells which can replace the olfactory receptor neurons during their continuous turn-over. The constant replacement of olfactory receptor neurons, make the olfactory mucosa “leaky” and thereby improving the nose-to-brain transport [30]. From these olfactory receptor neurons, axons project through the cribriform plate which separates the nasal and cranial cavities, on to mitral cells in the olfactory bulbs. Subsequently the olfactory bulbs will project on to different brain regions including the olfactory tract, the anterior olfactory nucleus, the piriform cortex, the entorhinal cortex, the amygdala, and the hypothalamus [31,32]. Upon intranasal administration, intra- and perineural transport is possible along these projections.

**Figure 1:**
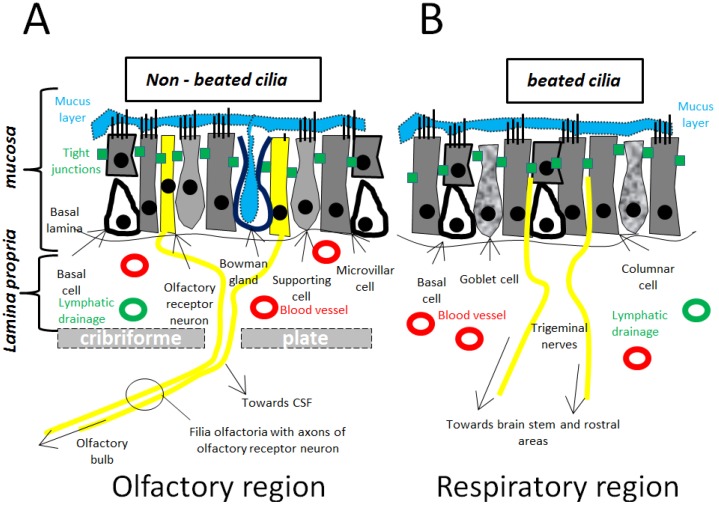
(**A**) Microscopic view of the olfactory mucosa, which represents ≤10% of the nasal cavity; (**B**) Microscopic view of the respiratory mucosa, which represents 80%–90% of the nasal cavity.

#### 3.1.2. Trigeminal Pathway/Respiratory Region (Figure 1B)

Another major player in nose-to-brain transport is the respiratory region in the nasal cavity. This area can reach up to 80%–90% of the nasal cavity in humans. This epithelium layer is necessary for warming and humidifying the inspired air, and for removing particles, allergens, and microorganisms. The layer consists of ciliated and non-ciliated columnar cells, goblet cells, and basal cells [33]. The goblet cells secrete mucus, which is propelled by the ciliated cells, towards the nasopharynx, where it is swallowed or expectorated. Interestingly, the respiratory region is innervated by projections of the trigeminal nerves. Also these nerves contribute to the nose-to-brain transport, as well-studied by Johnson *et al.* by applying a dye and following the transport along the trigeminal nerves [34]. The trigeminal nerve (or fifth V cranial nerve) has three main branches: the ophthalmic nerve (V1), the maxillary nerve (V2), and the mandibular nerve (V3). Only V1 and V2 will innervate the nasal passages via the ethmoidal branch (V1), nasopalatine branch (V2), and nasal branch (V2) [35]. From the respiratory epithelium, these branches will enter the brain at two sites, which is quite peculiar: the lacerated foramen, and the cribriform plate, thereby creating two entry sides into both the caudal and the rostral brain regions. In summary: when a pharmacological agent is administered intranasally, the agent can travel along the olfactory and trigeminal pathways, projecting towards the more rostral and more caudal, regions respectively. Transport along the nerves, either olfactory, or trigeminal, is believed to be perineuronal and intraneuronal, as discussed later.

#### 3.1.3. Other Possible Pathways (Figure 1A,B)

Perineural transport along the olfactory and trigeminal nerves is probably the major determinant of the nose-to-brain pathway. However, other connections between the nasal cavity and the CNS are also possible candidates. It is not unlikely that, for instance, the facial nerve or the Grueneberg ganglion are also entry points towards the CNS [36]. Beside the neural pathways, the vasculature pathways are also gaining interest. The olfactory region’s vascularization originates from small branches of the ophthalmic artery, while the respiratory region receives blood supply from branches of the maxillary artery [37]. Intranasally administered drugs can reach the systemic circulation via this vascularization and pass the BBB to enter the brain, especially if the applied drugs are small and lipophilic. More likely, molecules can also travel perivascularly along the channels associated with blood vessels, located between the outermost layer of blood vessels and the basement membrane of surrounding tissue [38]. Perivascular transport is not only driven by diffusion but also by bulk flow and arterial pulsation, which might explain the rapid distribution in the CNS of intranasally administered drugs [39]. Direct transport from the nasal cavity to the cerebrospinal fluid (CSF) has been reported, but is a rather unclear mechanism [40,41]. Absorption of the applied substance in the lymphatic vessels, located just under the basal lamina and draining the deep cervical lymph nodes of the neck, has also been reported [42].

### 3.2. Microscopical Anatomy (Figure 1, Figure 2)

In this paragraph, the hurdles and entry points that need to be overcome for an intranasally applied substance, to travel along the proposed routes to the CNS will be discussed.

**Figure 2:**
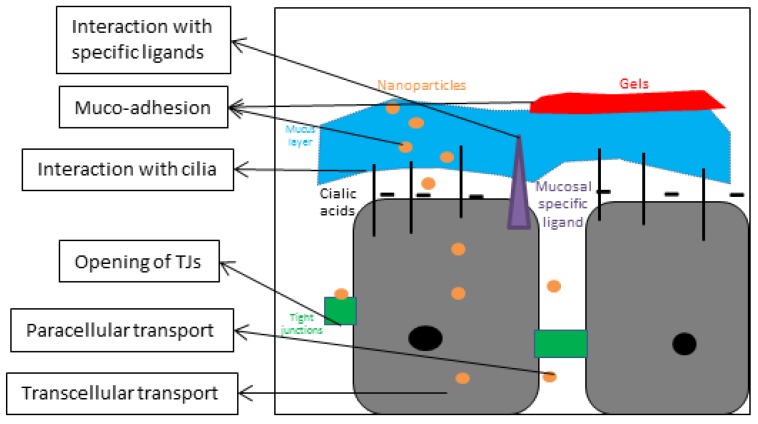
In-depth microscopic view of how formulations can further enhance the nose-to-brain pathway.

#### 3.2.1. Nasal Mucosa and Mucus

The first barrier that a pharmacological agent will encounter upon intranasal administration is the mucus layer covering the olfactory and respiratory mucosa. Mucus is a complex mixture secreted by the goblet cells in the mucosa, and consists out of 95% water, 2% mucin, 1% salt, 1% albumin, lysozymes, lactoferrin, immunoglobulines, and lipids [43]. The resulting pH of mucus in the nasal cavity is close to neutral or slightly acidic (pH 5.5–6.5) [44]. The mucus layer is propelled towards the pharynx by the cilia. It should be noted that only the cilia on the respiratory mucosa can move the mucus because the cilia on the olfactory mucosa lack the dynein arms which are necessary for motion [45]. These cilia can beat with a frequency of 1,000 beats per min, and propel the mucus 5 mm per min [46]. If a pharmacological agent successful reaches the nasal cavity, this is the first barrier to cross. Next, the drug can travel between cells, paracellularly or through cells, *i.e*., transcellularly.

#### 3.2.2. Tight Junctions (TJs)/Paracellular Transport between Nasal Mucosa

When the applied substance needs to travel between epithelial cells, it will have to cross several barriers. Two epithelial cells can be in close approximation with each other using several junctions: TJs, adhering junctions, desmosomes, and gap-junctions [47]. The intactness of these junctions will determine the success of the paracellular transport. There is also a size limitation: the hydrophilic channel between two epithelial cells is about 8 Å [47]. Questions about the integrity of these junctions remain, due to the constant renewal of the olfactory receptor cells, and the integrity of the entire mucosa [48]. Certain formulations can temporarily open these junctions and therefore promote nose-to-brain transport, as discussed later. This transport route is rapid and can occur within 30 min after application.

#### 3.2.3. Endocytosis/Transcellular Transport across Nasal Mucosa

An applied substance larger than 20 nm is believed to travel transcellularly. Many possible mechanisms are described in the literature and depend on the nature of the substance: clathrin-dependent or independent, caveolae-dependent or independent, macropinocytosis or phagocytosis [49]. It is reported than substances smaller than 200 nm prefer caveolae-mediated endocytosis, while substances in a range of 200–1,000 nm prefer clathrin-mediated endocytosis. Transcellular transport is generally rather slow, ranging from hours to several days. Substances entering an olfactory receptor neuron will undergo intraneuronal transport in the anterograde direction towards the olfactory bulb [50,51].

#### 3.2.4. Organization Nerves/Filia Olfactoria (Figure 1A)

In the lamina propria, just underneath the olfactory mucosal layer, the different axons of olfactory receptor neurons conjoin and are ensheathed by Schwann cells. These structural organizations are called filia olfactoria, and were first described by de Lorenzo *et al*. [52]. Typically 20 axons are bundled together in fascicles. One Schwann cell can easily ensheathe 5–10 fascicles, and thereby contain >100 axons. Perineuronal channels of 10–15 nm are present here and act as ionic reservoirs. Mesaxons are also present within the filia olfactoria and are pores that allow the passage of extracellular fluids. Transneuronal transport is dependent of the diameter of the axons, which in human ranges from 100 nm to 700 nm [53].

## 4. Pre-Clinical and Clinical Evidence of Nose-to-Brain Transport for GBM

In this paragraph, an overview is provided of the intranasally applied non-formulated substances relevant for the treatment of GBM (Table 1). Other non-formulated substances that have been already extensively reviewed fall beyond the scope of this manuscript [13,17,26,54]. Next, the enhancement by means of formulations for the intranasal transport will be discussed.

**Table 1:** Intranasal administration of pharmacological agents for the treatment of GBM, both in animals and in humans (+AZA: addition of acetazolamide for reducing CSF turnover).

Compound	Intranasal dose	Plasma concentration	CSF concentration	GBM model	Efficacy	Ref.
*Animals*						
Methotrexate	3.2 mg/kg	345 ± 58 ng/mL	1,278 ± 393 ng/mL	-	-	[55]
Methotrexate	2.5 mg	1 µg/mL	12.54 ± 1.54 µg/mL (+AZA)	9L rat glioma	Decreased tumor weight	[56]
5-fluorouracil	26.7 nmol	2.4 fmol/mL	6 fmol/mL (+AZA)	-	-	[57]
GRN163	0.65 µmol	-	-	U-521 MG rat glioma	Increased median survival from 35 days to 75.5 days	[58]
Vascular Stomatitis Virus	2.5 × 10^7^ PFU	-	-	U87 MG glioma	Selective infection and killing of olfactory bulb tumor	[59]
Neural Stem and Progenitor cells	3 × 10^5^ cells	-	-	U87 MG, NCE-G55T2, GL261	Rapid, targeted migration of cells towards intracerebral glioma	[60]
*Human*						
Monoterpene perillyl alcohol	440 mg/day	-	-	Recurrent GBM patients with at least 3 relapses	Increased median survival from 2.3 to 5.9 months	[61]

### 4.1. Intranasal Administration for GBM

#### 4.1.1. Animal Models

Given the advantages of nose-to-brain transport, reports have been made on treating GBM with intranasally applied substances. The most evident choice of substances is probably the intranasal administration of chemotherapy. Wang *et al*. reported the uptake of methotrexate into the CSF upon intranasal administration [55]. After intranasal or intravenous administration of methotrexate, the CSF and plasma were collected. They reported that intranasal administration delivered more methotrexate to the CSF and less to the plasma than intravenous administration. Methotrexate is a folic acid antagonist and is being used for a variety of systemic malignancies. Intravenous injections of methotrexate were attempted to treat malignancies of the CNS. Methotrexate is poorly lipophilic, highly bound to proteins in the serum, and can therefore barely pass the BBB [62]. Nevertheless, methotrexate is a very potent treatment modality and can also be used for CNS lymphoma via intrathecal injections [63]. Adverse events such as progressive paraplegia, anemia, and cerebral metabolite changes are not rare following intrathecal injections. Based on the positive findings of Wang *et al*., intranasal delivery of methotrexate was further investigated by Shingaki *et al*. [56]. This group inoculated 9 L rat glioma cells in the right frontal cortex of rats, followed by intranasal administration of methotrexate. They observed a significant antitumor response to intranasal methotrexate delivery. After 10 days and three administrations, the tumor weight was significantly lower in the rats that received intranasal methotrexate. The group that received intraperitoneal injections of methotrexate displayed only a small decrease in tumor weight.

Nose-to-brain transport of another chemotherapeutic agent, 5-fluorouracil, was also reported by Sakane *et al*. [64]. Comparing the plasma and CSF upon intravenous or intranasal administration brought them to the conclusion that the delivery of 5-fluorouracil in the CSF is augmented by nasal drug application. 5-Fluourouracil is a chemotherapeutic agent that is clinically used to treat breast cancers, melanomas, and pancreatic cancers. It is a pyrimidine analog, and will irreversibly inhibit the thymidylate synthase enzyme [65]. Side effects of systemic administration include myelosuppression, diarrhea and dermatitis. A similar study was performed with raltitrexed, which is clinically used in the treatment of advanced colorectal cancer and also inhibits the thymidylate synthase [66]. After intranasal administration they found different concentrations of raltitrexed in the brain, with rank order: olfactory bulbus > olfactory tract > cerebrum > cerebellum. Intranasal administration delivered significantly more raltitrexed into the CNS than intravenous administration. These results are encouraging, and suggest that some chemotherapeutics that were put aside due to poor BBB permeability might have to be reconsidered using intranasal administration.

Not only are chemotherapeutics intranasally applied in the context of GBM, but also antisense oligonucleotides. An excellent study by Hashizume *et al*. provided evidence that antisense oligonucleotides can also travel from nose-to-brain and have therapeutic effects in a rat glioma model [58]. The compound, GRN163, is an antisense oligonucleotide targeting telomerase, which is expressed in a majority of GBM [67]. U-251 MG tumor cell bearing rats were treated with intranasal administration of GRN163. After only 30 min, they tracked the compound in the trigeminal nerves and the brain stem, suggesting rapid distribution. Treatment for 12 consecutive days, starting when a 20 mg tumor was already present, resulted in a highly significant improvement of survival. Another interesting finding is the tumor specificity. They reported a preferential distribution of the compound in the tumor cells, which are positive for telomerase, whereas the normal tissue does not express telomerase. The preferential distribution seemed to be even more pronounced after intranasal administration than with CED administration [68].

Whereas chemotherapeutic agents cannot distinguish between GBM and healthy CNS, compounds as the GRN163 can be specific. Another example of tumor-tropism is the use of oncolytic viruses. These viruses preferentially proliferate in tumor cells and cause a lethal infection of these cells [69]. Özduman *et al.* provided evidence for the ability of the vesicular stomatitis virus VSVrp30a to destroy several human and mouse tumors implanted in the mouse brain, after intravenous injection of the virus [59]. Normally, the BBB will not permit the VSVrp30 to cross, and therefore, the intravenous injection would have no effect. However, they observed that upon tumor engraftment, the BBB becomes leaky and the virus can reach the CNS. Interestingly, Özduman also reported that when U87 glioma cells were stereotactically unilaterally engrafted on the olfactory bulbs of SCID mice, an olfactory bulb glioma was established. When the VSVrp30 was administered intranasally, the olfactory bulb gliomas were selectively infected and killed.

Nose-to-brain transport does not seem exclusively reserved for small molecules and viruses: a recent study by Reitz *et al*. showed the potential of cells to travel along the proposed route of transport [60]. In this research they managed to intranasally administer neural stem and progenitor cells (NSPCs). When mice were challenged with intracranial injections of U87, NCE-G55T2 or GL261 glioblastoma tumor cells, the intranasally administered NSPCs travelled specifically towards the tumor environment. The restorative potential and inherent pathotropism, in combination with guidance of danger signals, should explain the specific homing of the NSPCs towards the tumor environment. These NSPCs are a good candidate for the targeted delivery of biologically active gene products, both after intracerebral injection and after intranasal administration [70]. Therefore a clinical study (NCT01172964) has started with the intracerebral injection of the HB1.F3 neural stem cell line that was genetically modified and carries the prodrug-converting enzyme cytosindeaminase, which can convert the non-toxic prodrug 5-fluorocytosine to 5-fluorouracil. Given the new insights that neural stem cells can also be delivered intranasally, a non-invasive alternative is established.

#### 4.1.2. Clinical Setting

To our knowledge, there is only one clinical study concerning intranasal administration of chemotherapeutics in GBM patients. Da Fonseca *et al*. established a phase I/II study with the intranasal administration of monoterpene perillyl alcohol (POH), a Ras-protein inhibitor, in patients with a recurrent GBM [61,71]. At first, this study was initiated with an oral delivery of POH. However, serious adverse events of nausea, vomiting and diarrhea were reported. Upon reconsideration, an intranasal formulation was created, suited for nose-to-brain transport. In a small cohort of patients, they observed that the compound POH is well tolerated and that in some patients tumor regression is noticeable, suggestive of the antitumor activity of POH [71,72]. In the phase I/II study, they observed a significant increase in survival of recurrent primary GBM patients, from 2.3 to 5.9 months, compared to historically matched controls. A better response to treatment was noticed in patients with recurrent primary GBM in a deep location than in a lobar location. Next, a larger increase in survival was noticed in patients with a recurrent secondary GBM, progressing from a lower grade lesion, than in recurrent primary GBM patients. This means that patients evolving from a lower grade malignancy respond better to the intranasal POH, although secondary GBMs might have a slightly better natural prognosis as compared to primary GBMs.

#### 4.1.3. Possible Pitfalls

Despite all the promising accumulating (pre-) clinical data about the challenging nose-to-brain pathway, pitfalls are present and should be considered before attempting to validate this approach. Firstly, nose-to-brain transport is for now restricted to potent molecules. These molecules could be dissolved or dispersed in a small volume of liquid: the maximal delivery in mice is 24 µL, in rats 40–100 µL, and in humans 0.4 mL or formulated as a powder. Next, the applied substances have to resist the mucociliary clearing on the nasal mucosa, which transports the mucus at a rate of 5 mm/min. Although intranasal delivery can bypass the first pass effect in the liver, nasal cytochrome P450, as well as proteases and peptidases, are also present in the nasal mucosa, and can induce a pseudo-first-pass-effect. The cytochrome P450 can even have up to a fourfold higher NADPH-cytochrome P-450 reductase content than in the liver [73]. Furthermore, the translation of animal data to humans should be handled with caution. The anatomical differences between animal models and human are distinct. Rodent are obligatory nasal breathers, while primates are oronasal breathers. The nasal passage in rat is more complex than in humans, and has a larger surface-to-volume ratio. Nasal cavities in mice, rat and human present a volume of 0.032, 0.26, and 25 cm³, respectively [33]. The differences in anatomy and physiology can also be beneficial: CSF replacement in humans takes about 5 h, while in mice only 1.5 h. The slow CSF replacement is even more pronounced in older humans, which represent the dominant GBM patient population [33]. For these reasons, and to further increase the efficacy and potential of nose-to-brain transport, formulations can be developed. Pharmaceutical formulations can offer the active compound stability in its environment of administration, protection against possible destruction, and even specificity for the targeted tissue. These features should result in an increased half-life time, and concentration in the CSF, of the active compound and therefore an increased pharmacological effect.

## 5. Improvement of the Nose-to-Brain Pathway through Formulations

Many types of formulations can be developed according to the requirements of their application (Table 2). In the case of formulations for intranasal administration, the uptake of active molecules in the brain is mainly formulated as nanoparticles (Figure 2). Nanoparticles are defined as having a size smaller than 1 µm. They are designed to protect the drug, and transport them transcellularly, or paracellularly, depending on their properties, to the CNS. In the first paragraph of this section, the potential of nano-technology with different polymers and lipids will be underlined. Next, the application of ligand-specific lectins, emulsions and gels, which are used to increase the nose-to-brain transport, will also be discussed. Finally, several indirect enhancers of the intranasal pathway will be discussed.

### 5.1. Via Nanoparticles

#### 5.1.1. Polymer-Based Nanoparticles

Chitosan (CS) is a β-(1–4)-linked d-glucosamine and *N*-acetyl-d-glucosamine co-molecule, which represents a linear backbone structure linked through glycosidic bonds. CS is obtained upon the deacetylation of chitin, derived from crustacean shells. This molecule contains primary amines which can be protonated, and are positively charged in most physiological fluids. CS is, in many aspects an interesting polymer in which active molecules can be packaged for intranasal transport, and is therefore one of the most studied polymers in the field of transmucosal drug delivery [74,75]. This excipient is known as a polycationic, biocompatible, and biodegradable polymer, which presents mucoadhesive and permeation-enhancing properties and which presents non toxicity and low immunogenicity [76]. In intranasal delivery, it improves the nasal residence time of the formulation by decreasing the mucociliary clearance due to its bioadhesive properties [77,78]. This excipient could be used to elaborate different types of intranasal formulations including solution, dispersion, and powder formulation. CS based intranasal powder has been shown to possess a higher residence time than a solution [79]. Moreover, CS has the property to transiently open the tight junction of the mucosal epithelium, which increases the permeability of very polar compounds such as peptides, proteins or nucleic acids [80]. CS nanoparticles can be prepared according to several methods, as reported in the excellent review of Amidi *et al*. [80]. The most popular method for intranasal administration is ionic gelation, in which an anionic solution is added drop wise to the polycationic CS and crosslinks, *i.e*., performs gelation, to form nanoparticles [81]. A characteristic of the CS nanoparticles is their positive charges in acidic to neutral pH, resulting from the primary amines (pKa ~ 6.5). In physiological pH, the sialic acids and ester sulfates (pKa ~ 1.0–2.6) in the mucus layer are strongly negatively charged, thereby the CS nanoparticles and the sialic acids and ester sulfates can form strong electrostatic interactions [44].

**Table 2:** Overview of the pharmacological formulations, both polymer based and lipid based, that increase the efficacy of nose-to-brain transport after intranasal administration.

Formulation compound	Structure	Formulation
*Polymer based*		
Chitosan (CS)	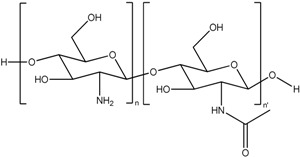	*Nanoparticle*
		[82,83,84,85,86,87]
		*Gel*
		[88,89,90]
Maltodextrin	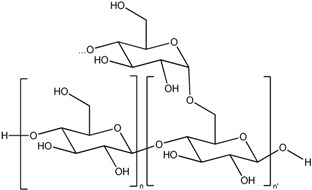	*Nanoparticle*
		[91]
Poly ethylene glycol (PEG)	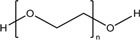	*Nanoparticle*
		[92,93,94,95,96,97,98,99,100,101]
		*Gel*
		[102]
Poly lactic acid (PLA)	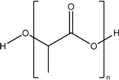	*Nanoparticle*
		[93,96,97,98,99,100]
Polylactic-co-glycolic acid (PLGA)	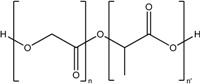	*Nanoparticle*
		[103,104]
PAMAM dendrimer	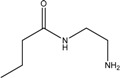	*Nanoparticle*
		[89,105]
Poloxamer	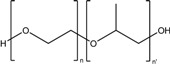	*Gel*
		[106]
*Lipid based*		
Glycerol monocaprate (CapmulTM)	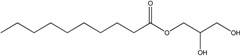	*Emulsion*
		[107,108]
Mixture of mono-, di-, and triglycerides and mono- and di- fatty esters of PEG (LabrafilTM)	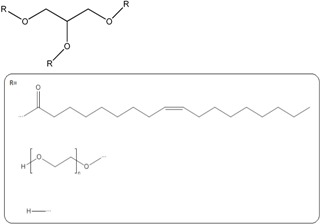	*Emulsion*
		[109]
Palmitate	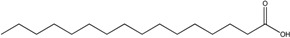	*Solid lipid particles*
		[110]
Glycerol monostearate	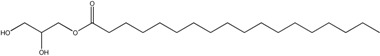	*Lipid particles*
		[111]
Phospholipids	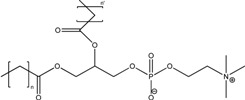	
		*Lipid nanovesicles*
		[112]
		*Liposomes*
		[113,114,115,116]

A first illustrative study was performed by Wang *et al*. They prepared estradiol containing CS nanoparticles with a mean size of 269.3 ± 31.6 nm and a zeta potential of +25.4 mV [82]. The charge of the particles is important in terms of stability: particles that display a charge > +20 mV are more likely to remain stable in solution [82]. Achieving high concentrations of estradiol in the CNS could be beneficial in treating Alzheimer’s disease [117]. CS nanoparticles were obtained by ionic gelation with tripolyphosphate anions. Estradiol was administered in a dose of 0.48 mg/kg either intranasally or intravenously. The CSF concentration was increased after intranasal administration from 29.5 ± 7.4 ng/mL for intravenous administration to 76.4 ± 14 ng/mL. A similar experiment was performed by Al-Ghananeem *et al*. [83]. In this study didanosine was incorporated into the CS nanoparticles. Here they also observed an increase of the didanosine, both in the CSF and in the brain, after intranasal administration of the formulated drug. A third study by Alam *et al*. incorporated thymoquinone into nanoparticles with a mean size of minimum 172.4 ± 7.4 nm, and a charge of +30.3 ± 2.15 mV [84]. This molecule has been proven to ameliorate cognitive deficits and neurodegeneration and therefore it might be of value in treating Alzheimer’s disease [118]. With a 18-fold increase of the brain-targeting efficiency and two-fold increase of the brain drug direct transport percentage as compared to thymoquinone in solution, *i.e*., without CS nanoparticle formulation, they concluded that the formulated thymoquinone had better brain targeting efficiency. Similar results were obtained by Fazil *et al*. [85]. They encapsulated another Alzheimer’s disease drug, rivastigmine, in CS nanoparticles, with an average size of 185.4 ± 8.4 nm and charge of +38.40 ± 2.85 mV. Fazil *et al*. coupled the particles with ROD-123, fluorescent rodamine dye, and observed a higher intensity of fluorescence in the brain upon intranasal administration compared to intravenous administration. Consequently, the concentrations of rivastigmine delivered by the formulated nanoparticles were significantly higher compared to intranasal or intravenous administration of rivastigmine in solution. The area under the curve (AUC) of intranasally administered rivastigmine CS particles in the brain was 3.11 times higher than intravenously administered rivastigmine in solution, and 1.92 times higher than intranasally administered rivastigmine in solution. These results suggest that after intranasal administration, the CS-rivastigmine particles reach the brain through both a direct nose-to-brain pathway, and the systemic circulation. Another example was published by Md *et al*. and provides supplementary evidence that chitosan nanoparticle formulations can improve nose-to-brain transport [86]. In this publication, bromocriptine was loaded into CS nanoparticles, resulting in particles with a mean size of 161.3 ± 4.7 nm, and a zeta potential of +40.3 ± 2.7 mV. Bromocriptine acts as a protector of dopaminergic cells and is therefore a well-known drug in Parkinson’s disease [119]. Bromocriptine was labeled with technetium, a radio-active substance, to measure the distribution. The brain/blood ratio was 0.47 ± 0.04 for intranasal administration of bromocriptine in solution, 0.69 ± 0.031 for intranasal administration of bromocriptine-CS nanoparticles and 0.05 ± 0.01 for intravenous administration of bromocriptine-CS nanoparticles. Interestingly, the increased concentration of bromocriptine in the brain was also reflected in clinical responses. Mice were administered haloperidol, which elicits typical Parkinson symptoms such as catalepsy and akinesia. These symptoms were reversible after bromocriptine-loaded CS nanoparticles administration. Rather than using chitosan to form nanoparticles, other nanoparticles can also be covered by CS chains, and thereby benefit from the advantages of CS. CS surface modifications of polystyrene particles resulted in an increased transmucosal transport [87].

Not only CS nanoparticles are studied for nose-to-brain transport, but also other polymers. For instance, maltodextrin has been used [91]. When these 60 nm nanoparticles (Biovector) were applied together with morphine, the duration of the antinociceptive activity was increased. With co-administration of the nanoparticles, no increase in morphine concentration in the blood was observed, and the effects of morphine were reversible by naloxone. Another extensively studied polymer is polyethylene glycol (PEG). This polyether structure has been shown to be very versatile in many applications, with a low toxicity. The addition of PEG onto the surface of nanoparticles, thereby improving the diffusion across the mucus, can improve their uptake [92]. Zhang *et al*. used methoxy PEG-polylactic acid (PLA) nanoparticles to improve the uptake of encapsulated nimodipine [93]. These PEG-PLA particles have a mean size of 76.5 ± 7.4 nm and a negative charge. The olfactory bulb/plasma and CSF/plasma nimodipine concentrations were significantly higher after nanoparticle formulation than for intranasal administration of nimodipine solution. Also, Wang *et al*. used the properties of PEG to slip molecules across the mucus barrier [94]. Indeed low molecular weight PEG, with a hydrophilic and almost neutrally-charged surface, has a minimized mucoadhesion. In this way, particles can rapidly slip through the mucus. They observed that polystyrene nanoparticles covered with low molecular weight PEG could slip faster through the mucus layer. This new insight results in an apparent paradox: should nanoparticles be strongly mucoadhesive (e.g., with chitosan), or should they slip across the mucus layer (e.g., with PEG coating)? Another interesting nanoparticle was created by Jain *et al*. [95]. These micellar PEG- based nanocarriers, only 23 nm in size, were loaded with zolmitriptan, a drug used for the treatment of migraine. The intranasal administration of the micellar formulation seemed superior to the intravenous administration and the intranasal administration of zolmitriptan in solution. Furthermore, a toxicological analysis for 28 days was also performed, with no signs of toxicity. Next, the potential use of poly lactic-co-glycolic acid (PLGA) polymer for the nose-to-brain pathway was explored by Seju *et al*. [103]. PLGA, like PEG, PLA and chitosan, is a biocompatible, biodegradable polymer and improves drug stability and release [120,121]. PLGA nanoparticles, with a size of 91.2 ± 5.2 nm, were loaded with olanzapine, an antipsychotic drug. Their poor bioavailability, due to the hepatic first-pass metabolism, and the poor brain uptake due to P-gp-efflux pumps, stimulated the search for an alternative administration route [122]. Formulating olanzapine in the PLGA nanoparticles increased the uptake into the brain by 6.35-fold after intravenous administration, and even 10.86-fold after intranasal administration. Also Md *et al*. further explored PLGA nanoparticles to further enhance the brain uptake, even after only intravenous administration. They observed that donezepil-loaded PLGA nanoparticles were superior in delivering donezepil to the brain [104]. A last kind of polymer which draws attention for nose-to-brain targeting, is the polyamidoamine (PAMAM) dendrimer. These polymers are repetitive branches that grow from a core. Many versatile molecules can be attached to their surface. Kim *et al*. connected an arginine onto the surface of a PAMAM dendrimer [105]. This resulted in nanoparticles with a size of 188.7 ± 1.9 nm and a charge of +22.3 mV. Small interference RNA (siRNA) targeting against the high mobility group box 1 protein (HMGB1) was electrostatically attached onto the nanoparticles. HMGB1 is released by dying cells and acts as a danger signal, thereby aggravating the damage of a stroke or other neurotoxic insults. Upon intranasal administration, they observed a wide distribution of the construct into the brain, including the hypothalamus, the amygdala, the cerebral cortex, and the striatum. Moreover, the localization of the PAMAM dendrimer and the siRNA was associated with an efficient knock-down of the protein of interest: HMGB1. When a stroke was induced into animals, the group that received the intranasal administration of the construct had a remarkably decreased infarction volume. Also using several behavioral tests, they could demonstrate that the treated group had a clear therapeutically response to the treatment.

#### 5.1.2. Lipid Based Nanosized Formulations

##### 5.1.2.1. Via (nano)Emulsions

In the literature, lipids are used as another possible method of formulating active compounds, more specifically lipophilic ones, in nanoparticles and further enhancing the nose-to-brain transport. Emulsions are a mixture of two or more liquids that are normally immiscible, such as oil-in-water. For intranasal transport, which requires small sizes, nanoemulsions, which contain droplets smaller than 100 nm, are a rising field of interest. Kumar *et al*. formulated risperidone, an antipsychotic drug, into a nanoemulsion [107]. The emulsion was made with Capmul MCM^TM^ as oily phase, polysorbate80 as surfactant, and distilled water as the aqueous phase. The mucoadhesivity of the risperidone nanoemulsion was increased with the addition of 0.5% CS (w/w) onto the droplet surface, which resulted in a globule size of 16.7 ± 1.21 nm. The superiority of the CS-coated nanoemulsion, in terms of brain/blood concentration ratio and more rapid transport, was demonstrated in comparison to the nanoemulsion without CS and in comparison to a simple risperidone solution, all after intranasal administration. Kumar *et al*. further studied this mucoadhesive nanoemulsion, with the incorporation of olanzapine, which is also an antipsychotic drug [108]. Here too, the mucoadhesive nanoemulstion seemed superior in terms of a higher drug targeting efficiency and direct nose-to-brain transport. Jogani *et al*. prepared a mucoadhesive microemulsion with tacrine, a reversible cholinesterase inhibitor used in Alzheimer’s disease [109]. For the emulsion, labrafil M 1944 CS^TM^ was used as an oily phase, cremophor as a surfactant, and distilled water as the aqueous phase. Mucoadhesive properties were prepared by the addition of carbopol 934 P^TM^ 0.5% (w/w) to the emulsion, resulting in globule size <27 nm and a net negative charge <−20 mV. The more rapid and more extensive transport of tacrine towards the brain showed the superiority of the mucoadhesive emulsion. Furthermore, mice were administered with scopolamine to induce amnesia. The mucoadhesive tacrine emulsion resulted in a faster regain of the memory loss.

##### 5.1.2.2. Via Solid Lipid Nanoparticles

Besides polymeric nanoparticles, lipid nanoparticles are also interesting candidates for brain targeting due to their rapid uptake by the brain, biocompatibility, biodegradability and weak toxicity. In this aspect, Eskanderi *et al*. formulated valproic acid in nanostructured lipid carriers based on palmitate. These lipid nanoparticles were prepared by solvent diffusion method followed by ultrasonication [110]. The brain/plasma ratio of valproic acid was 8.4 ± 0.32 after intranasal instillation, as compared to 1.65 ± 0.29 after intraperitoneal injection. Moreover, rats were better protected from seizures after intranasal administration. Joshi *et al*. formulated ondansetron HCl in solid lipid nanoparticles based on glycerol monostearate, which were also created by solvent diffusion [111]. Radiolabelling of these lipid nanoparticles showed a rapid uptake of these complexes in the brain of rabbits.

##### 5.1.2.3. Via Liposomes

Another classic drug delivery systems are liposomes. These structures consist of a lipid double layer that contains a hydrophilic core. Hydrophobic molecules can be integrated into the lipid layer and hydrophilic molecules can be encapsulated in the core. The use of liposomes is also feasible for intranasal administration, and nose-to-brain transport. Salama *et al*. encapsulated olanzapine in phospholipid based colloidal nanocubic vesicles for its brain targeting via the nasal route. The nanocubic vesicles were prepared by incorporating non-ionic copolymers, poloxamer 188 or 407^TM^, in the lipid bilayer [112]. These vesicles displayed a 37.9% absolute bioavailability and 100% brain targeting efficiency after intranasal administration. Arumugam *et al*. formulated rivastigmine in liposomes. Liposomes were formulated by the lipid layer hydration method using cholesterol and soya lecithin as lipid components [113]. When compared to rivastigmine in solution, the formulated rivastigmine-liposomes increased the rivastigmine concentration in the brain from 0.33 ± 0.29 µg/mL to 0.98 ± 0.74 µg/mL. The use of cationic liposomes was also explored by Migliore *et al*., who formulated ovalbumin with these liposomes [114]. Liposomes were prepared by combination of dioleoylphosphatidylcholine, cholesterol, and stearylamine. After intranasal administration, they observed a rapid distribution in the brain of the fluorescent labeled ovalbumin, with the highest concentration after 1 h. Priprem *et al.* also used liposomes for nose-to-brain transport. They formulated quercetin in liposomes, which consists of egg phosphatidylcholine and cholesterol followed by dispersion in 50% polyethylene glycol in water [115]. Rather than a quantitative approach, they observed an anxiolytic effect of intranasally administered formulated quercetin that was more rapid and at a lower dose as compared to oral administration.

### 5.2. Functionalization of the Nanoparticle Surface by Ligands

The previous paragraphs clearly demonstrated the potential of nanoparticles to improve and enhance nose-to-brain transport. Given good biological knowledge, skilled pharmaceutics, and an optimal control of the preparation process of these nanoparticles, a further improvement could be made by addition of lectins onto the surface of the nanoparticles. These lectins can recognize carbohydrate structures and thereby improve attachment to the mucus, cilia *etc.* This technique was well studied by Gao *et al*. with the conjugation of wheat germ agglutinin (WGA) onto PEG-PLA particles [96]. WGA has a specific binding to *N*-acetyl-d-glucosamine and sialic acids, both of which structures are abundantly present in the mucosa of the nasal cavity [123]. As a fluorescent marker, they included 6-coumarin into these particles and after intranasal administration they observed a two-fold increase in the brain of the WGA-conjugated particles, as compared to unmodified ones. Gao *et al*. then included vasoactive intestinal peptide (VIP) into the WGA-PEG-PLA nanoparticles [97]. Compared to intranasal administration of VIP in solution, the unmodified PEG-PLA particles increased the AUC in the brain 3.5–4.7-fold, and the WGA-modified PEG-PLA 5.6–7.7-fold. Liu *et al*. studied the toxicity and immunogenicity of the WGA-PEG-PLA construct and demonstrated its safety profile [98]. In a similar setting, Gao *et al*. also conjugated Ulex europeus agglutinin 1 (UEA1) to the PEG-PLA particles [99]. UEA1 can bind to L-fucose, located on the olfactory epithelium [124]. The conjugation of UEA1 led to a 1.7-fold increase in a fluorescent marker, 6-coumarin, in different brain areas in comparison to unmodified PEG-PLA nanoparticles. Chen *et al*. proved that the conjugation of solanum tuberosum lectin onto PLGA nanoparticles can increase the brain targeting efficiency 1.89–2.45-fold [125]. Park *et al*. explored the potential of conjugating low molecular weight protamine (LMWP) to PEG-PLA nanoparticles with a size of 110.77 ± 5.61 nm and charge of +2.42 ± 0.81 mV. LMWP works as a permeation enhancer with a good capacity for membrane translocation [100]. LMPW conjugation significantly increased the signal of the coumarin loaded onto the nanoparticles in the rat cerebrum, cerebellum, olfactory tract, and olfactory bulb compared to unmodified nanoparticles. Recently, lactoferrin was identified as an interesting protein. Lactoferrin is a natural binding iron protein, and its receptor is abundantly present on the respiratory epithelial cells and neurons [126]. This property was studied by Liu *et al*., demonstrating the enhanced nose-to-brain delivery of NAP, a neuroprotective peptide, when conjugating lactoferrin with PEG-co-poly(ɛ-caprolactone)nanoparticles [101]. By injection of β-amyloid, rats developed a model for Alzheimer’s disease, which was significantly improved by intranasal administration of lactoferrin-conjugated nanoparticles. To demonstrate the potency of these lectins, some examples are also available in the literature, in which a lectin is conjugated directly to the active molecule, not via a nanoparticle-intermediate as described before. In this way, Zhang *et al*. have established that the conjugation of cholera toxin B subunit can improve the brain-uptake of nerve growth factor (NGF) after intranasal administration. Cholera toxin B subunit binds to ganglioside GM1 in membrane raft microdomains on neural tissues. In fact, this is used for neuronal tracing of axonal pathways [127]. To induce an Alzheimer model in mice, they were challenged with β-amyloid. The increased concentrations of NGF by its conjugation with cholera toxin B could rescue these mice from the pathology, and improve spatial learning and memory abilities. A last approach in ligand-specific enhancement of the nose-to-brain pathway is the phage display method. This high-throughput screening method, allows the study of protein-protein interactions. Wan *et al*. identified nose-to-brain homing peptides [128]. The isolated phage reached the brain, after intranasal administration, at a rate 50-fold higher than a control phage. This is the first report that such a short peptide sequence can enhance nose-to-brain transport. Liposomes and nanoparticles can also be functionalized. Yang *et al*. described a formulation of rivastigmine in liposomes functionalized with a cell-penetrating peptide [116]. They observed higher concentrations of rivastigmine in the brain after intranasal administration of the cell-penetrating peptide-liposomes than with the liposomes or rivastigmine in solution.

### 5.3. Via Gels

From all the different polymers described in the previous sections, it is not only possible to make nanoparticles, but also mucoadhesive gels. Gels are three dimensional networks with a high viscosity, containing the active molecule. Charlton *et al*. demonstrated the gelling properties of chitosan and two low methylated pectins: LM-5 and LM-12 [129]. In a subsequent article, they published that the gel with 1% chitosan had a retention time on the human nasal mucosa that increased from 1.33 min to 12.58 min [88]. The potential of mucoadhesive gel formulations was also displayed by Perez *et al*. [89]. They coupled radioactive siRNA to PAMAM dendrimers to form dendriplexes, and formulated these particles into mucoadhesive gels containing either 1% (w/w) chitosan or 0.25% (w/w) carbopol 974P NF^TM^. These gels were prepared by blending the chitosan or carbopol with 23% (w/w) of thermosensible poloxamer to obtain *in-situ* gelation. Such a thermosetting gel has a phase transition below the temperature in the nasal cavity (32 °C to 35 °C) and above room temperature. Therefore it can be administered as a liquid. Different concentrations of the different gels were tested and no toxicity was observed. Two intranasal doses were necessary to achieve higher brain concentrations of radioactivity than achieved by intravenous administration of dendriplexes or intranasal administration of naked siRNA. Khan *et al*. prepared a gel with chitosan and hydroxyl propyl methyl cellulose and incorporated ropinirole, a dopamine D2 agonist [90]. Upon intranasal administration of the formulated ropinirole, concentrations in the brain were 8.5 times higher than after intravenous administration, and 3 times higher than after intranasal administration of non-formulated ropinirole. PEG, another popular polymer used for intranasal administration with or without mucoadhesive CS, was used by Zaki *et al*. to prepare a thermosetting gel with poloxamer conjugated with metoclopramide hydrochloride to enhance drug release [106]. Intranasal administration of the gel increased the bioavailability from 51.7%, for oral drug solution, to 69.1%. With similar gels, using carbopol, carboxymethyl cellulose and PEG, Babu *et al*. found an increased delivery of melatonin in the olfactory bulb [102]. Upon intranasal administrations of these gels, the increased delivery in the CSF was respectively 9.22-, 6.77- and 4.04-fold, as measured by microdialysis.

### 5.4. Via Indirect Enhancers/Devices (Figure 3)

There are also different approaches increasing the efficiency of nose-to-brain transport without using formulations. The route of transport is challenging, and not every obstacle can be overcome by the galenic formulations discussed in the paragraphs above. Therefore several indirect enhancers can be applied to further increase and optimize the transport to the brain. For instance, the inhibition of P-gp at the BBB can increase uptake in the brain. A portion of the intranasally applied substance can travel through the systemic circulation, into the brain. Graff *et al*. observed that co-administration of a P-gp inhibitor, rifampin, and an active substance, increased the brain concentration after nasal instillation [130]. Another possible method was proposed by Charlton *et al*. [131]. By applying a local vasoconstrictor, one could eliminate the uptake into the systemic circulation and thereby favor the nose-to-brain route. However, when applying ephedrine, no increased uptake was observed. This was in contrast with the results of Dhuria *et al*. [132]. They observed a significant increase in the brain, and decrease in the systemic circulation of hypocretin-1 after intranasal administration, in combination with phenylephrine administration. A possible explanation for this discrepancy suggested by Mistry *et al*., is that the rapid onset of action by phenylephrine is more advantageous than the long on-set of action of the ephedrine as used by Charlton *et al*. [54]. When an intranasally applied substance achieves high concentrations in the brain, a part will be rapidly transported and cleared by the turnover of the CSF. As suggested already in previous sections, the slower the CSF turnover is, the longer the active substance can remain in the CSF and be transported towards distinct brain regions. Therefore, Shingaki *et al.* tested the use of acetazolamide in combination with intranasal administration of 5-fluorouracil, given the previously demonstrated successes of 5-fluorouracil after intranasal administration [64]. Acetazolamide is a carbonic anhydrase inhibitor that can inhibit the secretion of the CSF in the choroid plexus and is clinically used to treat idiopathic intracranial hypertension [133]. They observed that the intravenous administration of acetazolamide can increase the nose-to-brain transport of intranasally applied 5-fluorouracil by 200%–300%.

The potential of nose-to-brain transport is now generally accepted and a number of non-formulated proteins and peptides, such as insulin and oxytocin are already being tested in humans [134,135]. The experiences from these trials, together with the classical trials that use the nose for e.g., vaccination, have stimulated private companies to develop several devices. These devices target powder or droplet substances specifically to the nasal cavity. In the study by Charlton *et al*., the superiority of drops over sprays was demonstrated [88]. Many interesting devices are entering the clinic and can serve as useful tools to implement nose-to-brain transport clinically in the healthcare industry. One example is the ViaNase^TM^ (Kurve technologies, Bothell, WA, USA). In a clinical study, insulin was administered to Alzheimer’s disease patients to improve their cognitive functions, given the neuroprotective properties of insulin [135]. The ViaNase^TM^ is a liquid based drug delivery device based on controlled particle dispersion. Other interesting devices are the OptiMist^TM^ (OptiNose, Oslo, Norway) and DirectHaler^TM^ (Lyngby, Denmark), which are devices that target liquid or powder nasal formulations to the nasal cavity, including the olfactory region, without deposition in the lungs or oesophagus. The OptiMist^TM^ is a breath actuated device and has been proven to result in more deposition in the nasal cavity than a spray pump [136,137]. With respect to clinical application, not only will the device be of importance but also the position of the head as it is likely that active substances might come loose from the nasal mucosa by gravity [138]. In the majority of the articles discussed, animals were placed in the supine position. Van den Berg *et al.* found that the supine position with the head at 70° and 90° was the most favorable [139]. In non-human primates, the most favorable position is the “praying to Mecca”, with the head down and forward [27].

**Figure 3:**
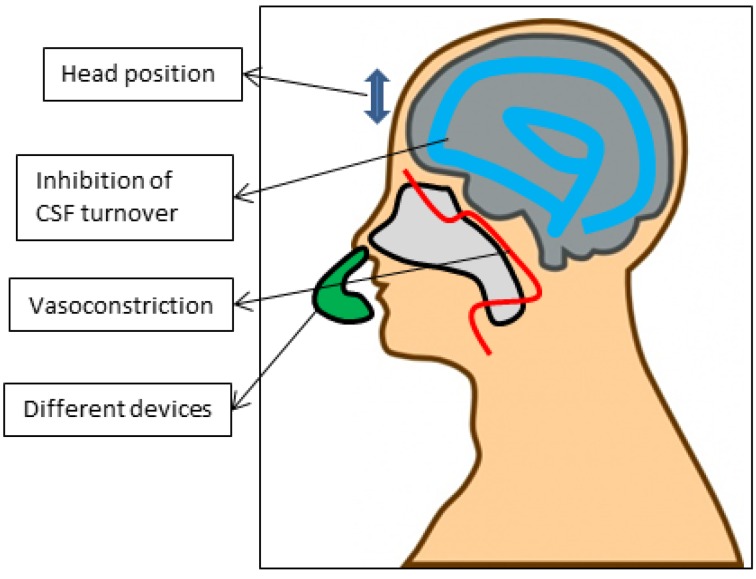
Factors that can influence the efficacy of intranasally administrated substances, with the focus on indirect enhancers.

## 6. Conclusions

As reported here, the nose-to-brain transport route holds great potential to treat GBM and many other brain diseases. It is only in recent years that the route of transport and the underlying mechanisms have begun to be understood. After intranasal administration, drugs can reach the CNS by traveling intra- and perineuronally along the olfactory and trigeminal nerves, the vasculature, and even the lymphatic system. In the context of GBM, a summary of pre-clinical and clinical studies in which the intranasal route is used for GBM treatment modalities was reported. These studies provide a future for intranasal administration in the treatment of GBM. Together with these new findings, the pharmaceutical sciences offer new formulations to overcome the hurdles inherent in treating this disease using the nose-to-brain route. Achieving therapeutical concentration in the CNS will be dependent on the formulation of the drug. Whether the drug needs to be encapsulated in nanoparticles, gels or emulsions will depend on the nature of the drug and the required delivery. These formulations will be necessary to enhance and optimize further the efficacy of the nose-to-brain pathway. Moreover, indirect enhancers will also be crucial for the ultimate success of intranasal administration. It is expected that in the following years, neurosurgeons, oncologists and neurologists will meet the pharmaceutical sciences. This cross-talk could lead to the development of a new therapy concept regarding the intranasal administration route, and might ultimately lead to a better prognosis for GBM and other brain diseases.
